# Oxoglaucine Suppresses Hepatic Fibrosis by Inhibiting TGFβ-Induced Smad2 Phosphorylation and ROS Generation

**DOI:** 10.3390/molecules28134971

**Published:** 2023-06-24

**Authors:** Bakhovuddin Azamov, Kwang-Min Lee, Jin Hur, Shakhnoza Muradillaeva, Wan-Seog Shim, Chanhee Lee, Parkyong Song

**Affiliations:** 1 Department of Convergence Medicine, School of Medicine, Pusan National University, Yangsan 50612, Republic of Korea; 2 Department of Life Science and Environmental Biochemistry, Life and Industry Convergence Research Institute, Pusan National University, Miryang 50463, Republic of Korea

**Keywords:** liver fibrosis, oxoglaucine, TGFβ signaling, collagen type 1, reactive oxygen species

## Abstract

Hepatic fibrosis is the first stage of liver disease, and can progress to a chronic status, such as cirrhosis or hepatocellular carcinoma. Excessive production of extracellular matrix (ECM) components plays an important role in the development of fibrosis. Mechanistically, transforming growth factor beta (TGFβ)-induced phosphorylation of Smad is thought to be a key signaling pathway in the development of liver fibrosis. Although the natural isoquinoline alkaloid oxoglaucine (1,2,9,10-tetramethoxy-7H-dibenzo(de,g)quinolin-7-one) exerts numerous beneficial effects, including anti-cancer, anti-inflammatory, and anti-osteoarthritic effects in diverse cell types, the effects of oxoglaucine on liver fibrosis and fibrogenic gene expression have not been fully elucidated. The aim of this study is to evaluate the signaling pathway and antifibrotic activity of isoquinoline alkaloid oxoglaucine in TFGβ-induced hepatic fibrosis in vitro. Using Hepa1c1c7 cells and primary hepatocytes, we demonstrated that oxoglaucine treatment resulted in inhibition of the expression of fibrosis markers such as collagen, fibronectin, and alpha-SMA. Subsequent experiments showed that oxoglaucine suppressed TGFβ-induced phosphorylation of Smad2 and reactive oxygen species (ROS) generation, without altering cell proliferation. We further determined that the increase in Smad7 by oxoglaucine treatment is responsible for the inhibition of Smad2 phosphorylation and the anti-fibrogenic effects. These findings indicate that oxoglaucine plays a crucial role in suppression of fibrosis in hepatocytes, thereby making it a potential drug candidate for treatment of liver fibrosis.

## 1. Introduction

Hepatic fibrosis is a consequence of extracellular matrix (ECM) deposition in response to chronic liver injury, and it contributes to an increased risk of hepatocellular carcinoma [1]. Various factors, such as non-alcoholic steatohepatitis (NASH), chronic HCV infection, non-alcoholic fatty liver disease (NAFLD), and autoimmune hepatitis are leading causes of liver fibrosis [2,3]. Due to chronic inflammation in all of these conditions, there is an excessive production of fibrogenic matrix, which can lead to liver cirrhosis. This is defined as a late stage of progressive fibrosis, with disruption of the physiological structure and function of the liver [4]. At the molecular and cellular levels, different signaling pathways and numerous cell–cell interactions affect liver fibrosis [5,6]. Various growth factors play crucial roles in ECM deposition and activation of hepatic stellate cells (HSCs), which are a major source of myofibroblasts in the fibrotic liver region. For example, platelet-derived growth factor (PDGF) signaling stimulates the Ras-MAPK pathway which, in turn, increases intracellular calcium levels and liver fibrosis [7]. Recent research has shown that angiogenesis contributes to the development of fibrosis during long-term liver damage. There is a positive relationship between vascular endothelial growth factor (VEGF) and liver fibrosis [8]. In this study, HSC activation was significantly inhibited in hepatocyte-specific *Vegfa*-deletion mice during HCC progression. Most importantly, transforming growth factor beta (TGFβ) is thought to be a master mediator of fibrogenesis [9,10]. TGFβ ligands bind to a heterotetrameric receptor complex comprising one of the seven type I receptors in combination with one of the five type II receptors [11]. Once the ligand-receptor complex has formed, activation of kinase activity results in phosphorylation cascades involving the transcription factor Smad2/3 [12,13]. Phosphorylated Smad2/3 accumulates in the nucleus, where it directly regulates the transcription of important profibrotic genes, such as collagen and fibronectin [14]. Additionally, TGFβ has been known to increase ROS generation, which plays a crucial role in stimulating liver fibrosis [15] As a negative-feedback regulator, Smad7 interferes with the interaction between TGFβ type I receptor and Smad2 [16,17]. Moreover, Smad7 can suppress TGFβ signaling in a type I receptor-independent manner [18].

Oxoglaucine is a phytochemical compound that belongs to the family of isoquinoline alkaloids, and it is found in various herbs, such as *Sarcocapnos baetica* and *Sarcocapnos saetabensis* [19]. Similar to berberine, which is another type of isoquinoline alkaloid, oxoglaucine has various biological effects, such as antifungal, anticancer, and antiplatelet activity [20,21,22]. Interestingly, previous studies have also implicated oxoglaucine in inflammation [23,24,25]. Remichkova et al. reported that oxoglaucine inhibits lipopolysaccharide (LPS)-mediated TNFα and IL-6 production in peritoneal macrophages [26]. Oxoglaucine also inhibits the expression of pro-inflammatory cytokines, including TNFα, IL-6, IL-1β, and MMP-13 in OA chondrocytes [27]. Finally, low doses (1 or 2 mg.kg^−1^) of oxoglaucine improved the outcome of *Klebsiella pneumonia* infection in vivo [28]. Although there is a strong relationship between fibrosis and inflammation [29], the potential effects of oxoglaucine on hepatic fibrosis, and the cellular mechanism by which oxoglaucine exerts its effect in murine hepatocytes, remain unclear. This study, therefore, investigated whether oxoglaucine suppresses TGFβ signaling and the expression of fibrogenic markers in Hepa1c1c7 cells and primary hepatocytes, in addition to investigation of the mechanisms responsible for oxoglaucine-mediated antifibrotic effects.

## 2. Results

### 2.1. Effect of Oxoglaucine on Cellular Viability

**Figure 1:**
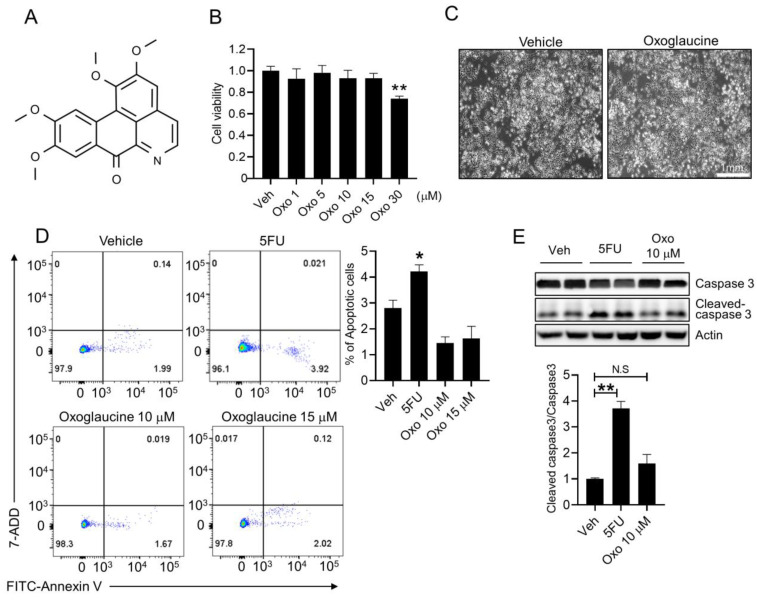
Determination of the non-toxic concentration of oxoglaucine in hepatocytes. (**A**) Chemical structures of oxoglaucine. (**B**) Survival of Hepa1c1c7 cells after dose-dependent oxoglaucine treatment for 24 h. (**C**) Light microscopy cell images were obtained after 24 h of 10 μM oxoglaucine treatment. (**D**) Apoptosis was analyzed by FACS assay using Annexin V/7-ADD double staining, following 24 h of oxoglaucine treatment. The mean for the total amount of apoptotic cells from four independent experiments is presented. (**E**) Cleaved caspase 3 levels were determined. Values are shown as ± S.E.M * *p* < 0.05 and ** *p* < 0.01. N.S indicates not significant.

The chemical structure of oxoglaucine is shown in Figure 1A. To verify the non-cytotoxic concentration of oxoglaucine, we performed a WST1 assay to assess mouse Hepa1c1c7 cell viability. A previous study showed that 20 μM oxoglaucine exhibited very low cytotoxicity in a liver carcinoma cell line [30]. Consistent with this research, there were no significant changes in Hepa1c1c7 cell viability up to a concentration of 15 μM (Figure 1B). However, 30 μM oxoglaucine reduced the cell viability. Light microscopy imaging further indicated that 24 h of exposure to 10 μM oxoglaucine did not induce any changes in the cell number or morphology (Figure 1C). In addition, we evaluated the proportion of apoptotic cells following oxoglaucine treatment by flow cytometry. As shown in Figure 1D, in contrast to 5-fluorouracil (5FU) treatment, which greatly increased the percentage of early and late apoptotic cells, the percentage of apoptotic cells was similar between the vehicle- and oxoglaucine-treated groups. Similar to this result, oxoglaucine did not increase cleaved forms of effector caspase 3 either (Figure 1E). Thus, we confirmed that 10–15 μM oxoglaucine did not affect hepatocyte viability.

### 2.2. Oxoglaucine Attenuates TGFβ-Induced Phosphorylation of Smad2 and Fibrogenic Gene Expression

**Figure 2:**
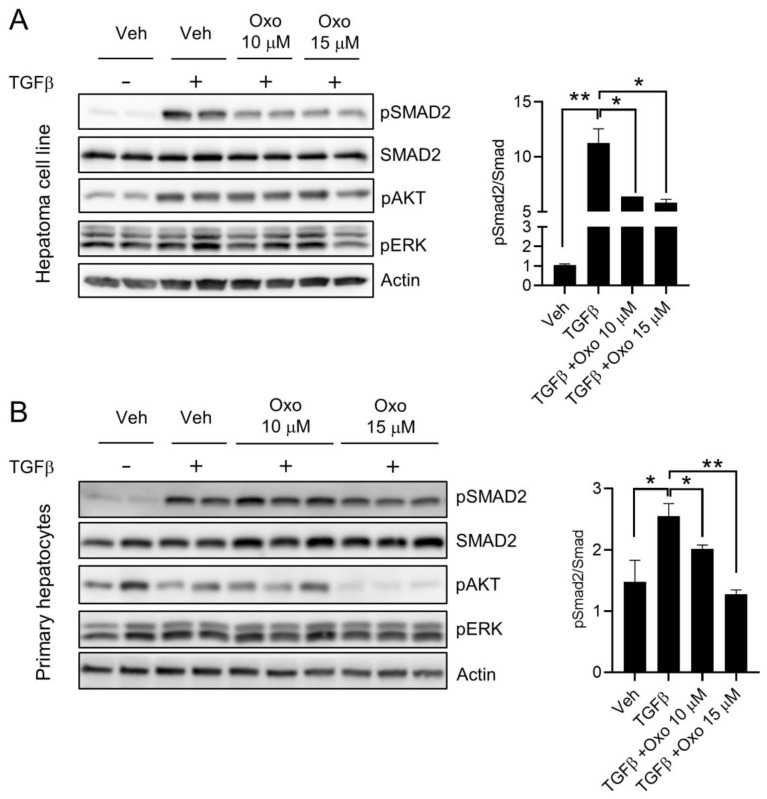
Effects of oxoglaucine pretreatment on TGFβ-induced Smad2 phosphorylation. Hepa1c1c7 cells (**A**) and primary hepatocytes (**B**) were treated with the indicated concentrations of oxoglaucine for 24 h, followed by TGFβ (5 ng/mL) stimulation for an additional 6 h. The lysates were subjected to immunoblot assay using various primary antibodies (left panel). The right panel shows quantification of the phospho/total Smad2. Values are shown as ± S.E.M * *p* < 0.05 and ** *p* < 0.01.

To investigate the effects of oxoglaucine on TGFβ signaling, Hepa1c1c7 cells and primary hepatocytes were pretreated with oxoglaucine for 24 h, then exposed to TGFβ for 6 h. We found that TGFβ-induced phosphorylation of Smad2 in Hepa1c1c7 cells was significantly inhibited (approximately 40% lower than in the cells treated only with TGFβ) by pretreatment with oxoglaucine, without any effect on the total Smad2 protein level (Figure 2A). Similar to Hepa1c1c7 cells, oxoglaucine also attenuated TGFβ-induced phosphorylation of Smad2 in primary hepatocytes (Figure 2B). Previous studies have demonstrated that TGFβ activates MAPK and PI3K signaling pathways in a context-dependent manner [31,32]. In our study, TGFβ treatment in Hepa1c1c7 cells increased AKT phosphorylation, whereas ERK phosphorylation remained similar to that in the vehicle group. However, the phosphorylation of AKT in Hepa1c1c7 remained unchanged after oxoglaucine treatment (Figure 2A). In marked contrast, AKT phosphorylation in primary hepatocytes on oxoglaucine treatment was significantly decreased (Figure 2B).

**Figure 3:**
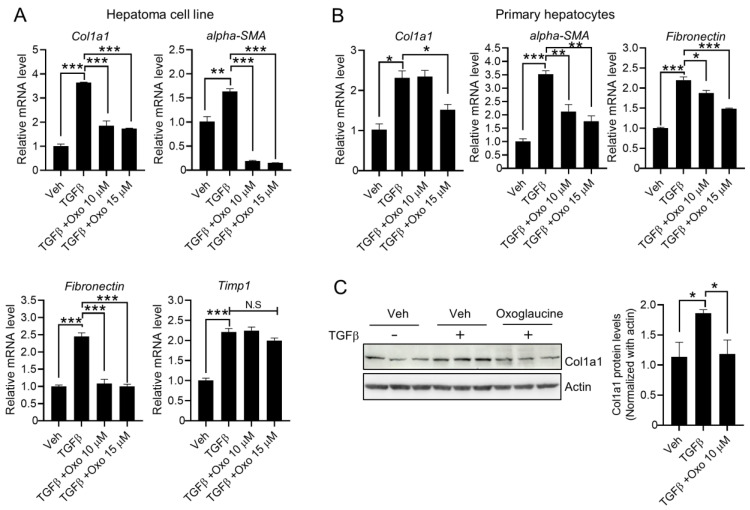
Oxoglaucine suppressed TGFβ-induced fibrogenic gene expression in hepatocytes. TGFβ (5 ng/mL)-induced fibrosis-related genes (*Col1a1*, *αSMA*, *Fn1*, and *Timp1*) were analyzed under oxoglaucine pretreatment conditions in Hepa1c1c7 cells (**A**) and primary hepatocytes (**B**) using qPCR. Each gene was normalized by *Gapdh*. (**C**) Protein levels of collagen type 1 alpha 1 after oxoglaucine treatment. Normalization was performed by actin. Values are shown as ± S.E.M * *p* < 0.05, ** *p* < 0.01, and *** *p* < 0.001. N.S indicates not significant.

Next, we investigated whether TGFβ-mediated fibrogenic gene expression was affected by oxoglaucine treatment. TGFβ primarily increases type I collagen, fibronectin protein, and gene expression, together with *αSMA*, to induce fibrosis [33]. Similar to the effect on Smad2 phosphorylation, exposure to oxoglaucine inhibited the transcript levels of fibrosis-related markers such as collagen type 1 alpha 1, *αSMA*, and fibronectin in Hepa1c1c7 cells (Figure 3A) and primary hepatocytes (Figure 3B). Meanwhile, there was no significant difference in *Timp1* mRNA levels (Figure 3A). Finally, pretreatment with oxoglaucine significantly inhibited the protein expression of collagen type 1 alpha 1 induced by TGFβ stimulation (Figure 3C), indicating that oxoglaucine can significantly suppress the fibrosis status in vitro. Moreover, treatment with oxoglaucine alone did not affect Smad2 phosphorylation or the mRNA levels of fibrogenic markers, except for *α-SMA* (Supplemental Figure S1A,B). Activated hepatic stellate cells (HSCs) also play a crucial role in liver fibrosis. Thus, we investigated whether oxoglaucine could regulate TGFβ signaling and expression of fibrogenic markers in human hepatic stellate cells. Similar to Hepa1c1c7, there were no significant changes in cell morphology or HSC viability (Supplemental Figure S1C). Although oxoglaucine pretreatment largely suppressed the TGFβ-induced collagen type 1 alpha 1 and alpha-SMA levels, phosphorylation of Smad2 was not affected by oxoglaucine in stellate cells (Supplemental Figure S1D).

### 2.3. Oxoglaucine Suppresses the mRNA Levels of Pro-Inflammatory Cytokines

**Figure 4:**
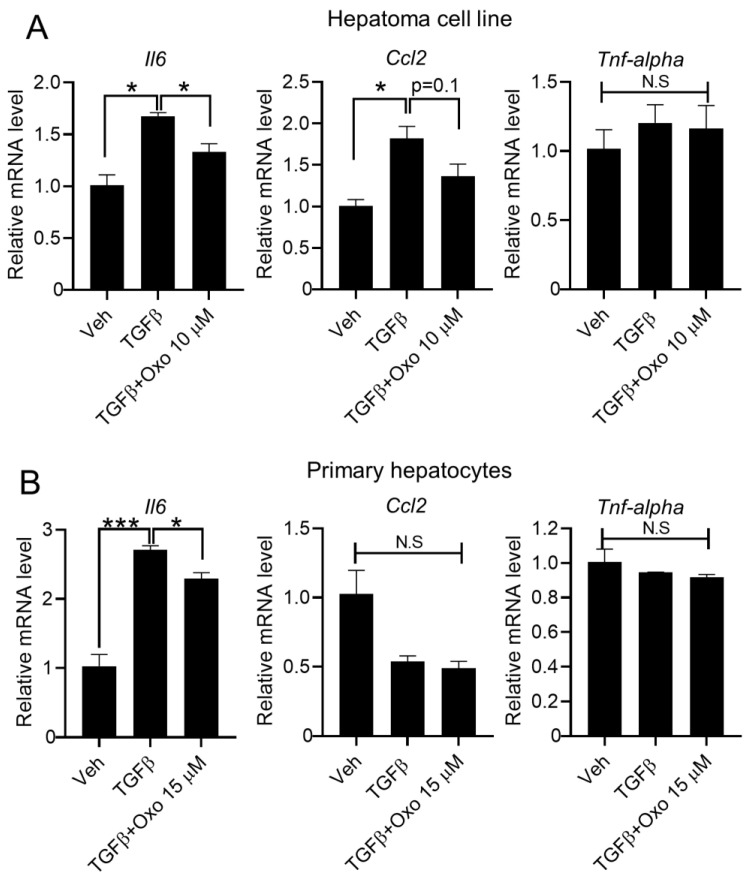
Oxoglaucine alleviated TGFβ-induced inflammatory cytokine expression. (**A**) Hepa1c1c7 cells and (**B**) primary hepatocytes were incubated with oxoglaucine for 24 h, followed by TGFβ stimulation, and the transcript levels of *Il6*, *Ccl2*, and *Tnfα* were then determined. Values are shown as ± S.E.M * *p* < 0.05 and *** *p* < 0.001. N.S indicates not significant.

Inflammation is a representative outcome of liver disease, and is associated with hepatic fibrosis and cirrhosis [34]. Importantly, TGFβ is a crucial modulator of T-cell homeostasis, and functions as an important link between immune and fibrogenic cells [34,35]. Therefore, we examined whether oxoglaucine affects the expression of pro-inflammatory cytokines. As shown in Figure 4A, TGFβ significantly increased *Il6* and *Ccl2* mRNA levels in Hepa1c1c7 cells. Interestingly, oxoglaucine treatment decreased TGFβ-induced *Il6* and *Ccl2* expression. However, mRNA levels of Tnf-alpha remained similar among all experimental conditions (Figure 3A). Next, we evaluated the expression of pro-inflammatory cytokines in primary hepatocytes. Only *Il6*was found to be induced by TGFβ stimulation, and the increased *Il6*levels were suppressed by oxoglaucine (Figure 4B). These results suggest that oxoglaucine blocks inflammation by reducing the levels of various inflammatory cytokines.

### 2.4. Effect of Oxoglaucine on ROS Levels in Hepatocytes

**Figure 5:**
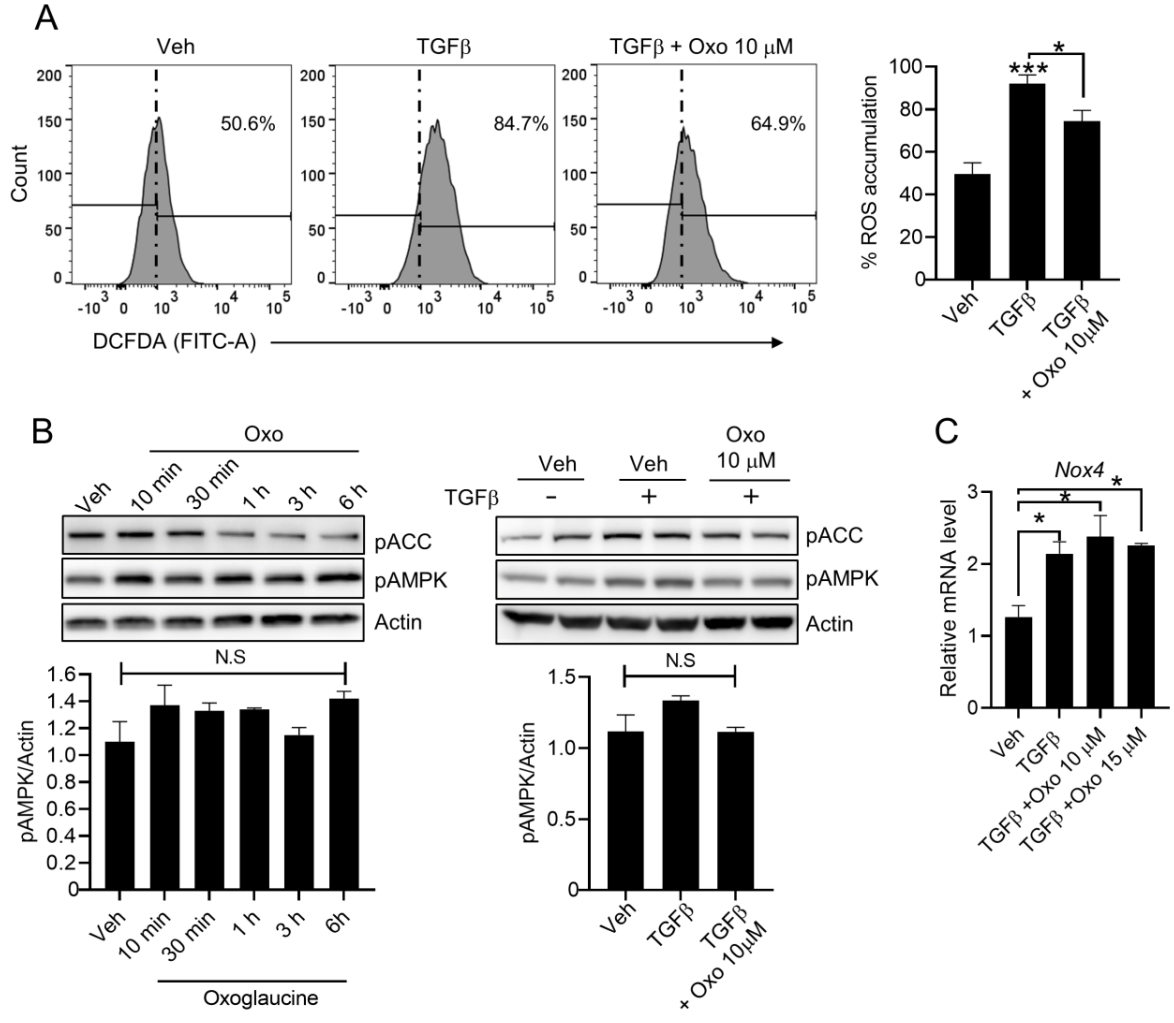
Oxoglaucine suppressed TGFβ-induced ROS production without affecting AMPK phosphorylation. (**A**) Measuring ROS levels using FACS analysis by DCFDA staining with indicated conditions. Results are represented as mean values from three independent experiments. (**B**) Hepa1c1c7 cells were incubated with oxoglaucine alone for the indicated time (left panel). Additionally, oxoglaucine was pretreated for 24 h, followed by TGFβ stimulation. AMPK activation was evaluated using anti-phospho-AMPK (pAMPK) and phospho-acetyl-CoA carboxylase (pACC) antibodies. (**C**) The NOX4 transcript levels were analyzed under the indicated condition. Values are shown as ± S.E.M * *p* < 0.05, and *** *p* < 0.001. N.S indicates not significant.

Reactive oxygen species (ROS) contribute to initiation and progression of TGFβ-mediated fibrosis [36]. Thus, we further investigated whether oxoglaucine could suppress TGFβ-induced cellular ROS production. Interestingly, pre-treatment of oxoglaucine resulted in lower the ROS levels (Figure 5A). Expectedly, oxoglaucine alone treatment did not significantly affect basal level of intracellular ROS (Supplemental Figure S1E). AMP-activated protein kinase (AMPK) is a highly conserved sensor of adenosine nucleotide levels that is activated when energy is depleted. AMPK regulates whole-body energy homeostasis by affecting various metabolic tissues, such as skeletal muscle, liver, and adipose tissues [37]. Since AMPK inhibits the TGFβ signaling pathway and ROS accumulation [38,39,40], we further investigated AMPK activation by oxoglaucine by measuring AMPK and its downstream target, acetyl-CoA carboxylase (ACC) phosphorylation. As shown in Figure 5B, oxoglaucine treatment, both with and without TGFβ, did not increase phosphorylation of AMPK and ACC. Previous studies also demonstrated that TGFβ increased production of ROS by regulating NADPH oxidase (NOX) levels in hepatocytes [36]. We confirmed that the transcript levels of NOX4, which involved in TGFβ-induced ROS generation, remained similar after oxoglaucine treatment (Figure 5C).

### 2.5. Suppression of Smad7 Blocks the Anti-Fibrogenic Effects of Oxoglaucine

**Figure 6:**
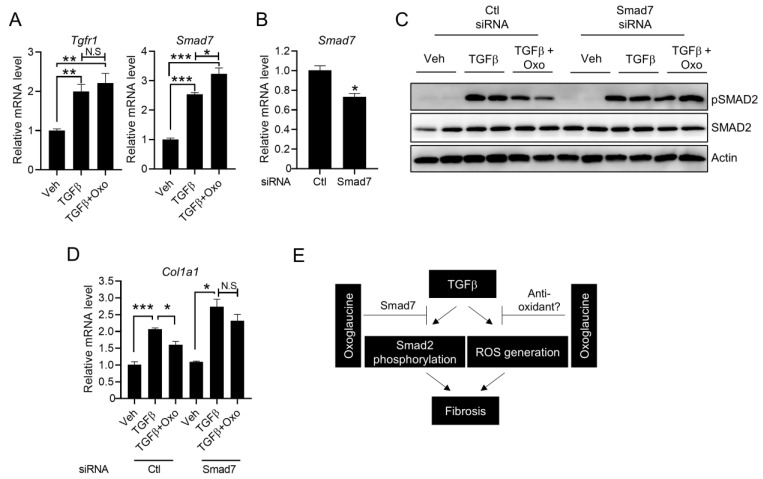
Oxoglaucine decreased collagen type 1 expression through Smad7. (**A**) qPCR analysis of TGFR1 and Smad7 mRNA expression under the indicated conditions. (**B**) Knockdown efficiency of Smad7 in siRNA-transfected cells. Hepa1c1c7 cells were transfected with Smad7 siRNA as indicated (50 nM final concentration). After 8 h, oxoglaucine was added for 24 h. Cell lysates were subjected to Western blot analysis to determine the level of Smad2 phosphorylation (**C**), and the mRNA level of collagen type 1 alpha 1 was determined by qPCR (**D**). (**E**) Working mechanisms illustrating the protective effects of oxoglaucine against TGFβ-induced fibrosis. Values are shown as ± S.E.M * *p* < 0.05, ** *p* < 0.01, and *** *p* < 0.001. N.S indicates not significant.

To determine how oxoglaucine affects TGFβ-mediated Smad2 phosphorylation and fibrogenic gene expression, TGF receptor expression was analyzed. In accordance with previous studies [41,42], TGFβ increased expression of the TGF type I (TβRI) receptor (Figure 6A). However, the transcript levels of type I (TβRI) receptors were not significantly affected by oxoglaucine treatment. Because Smad7 could inhibit the phosphorylation of Smad2, we further investigated whether oxoglaucine could modulate Smad7 transcript levels. Compared with TGFβ alone, pretreatment with oxoglaucine further increased Smad7 mRNA levels (Figure 6A). Next, to determine whether Smad7 is required for the effect of oxoglaucine, we performed silencing experiments. Based on the qPCR results, the knockdown efficiency was approximately 35% (Figure 6B). Importantly, the decrease in Smad2 phosphorylation by oxoglaucine treatment was largely attenuated when the cells were transfected with Smad7 siRNA (Figure 6C). As expected, the suppression of collagen type 1 alpha 1 mRNA levels by oxoglaucine was largely recovered under Smad7 silencing conditions (Figure 6D). Interestingly, there was a trend toward increased TGFβ-induced Col1a1 expression in Hepa1c1c7 cells when Smad7 siRNA was transfected. We speculated that TGFβ responsiveness could be further increased when the feedback inhibitor, Smad7, was silenced. Taken together, these results suggest that regulation of Smad7 by oxoglaucine is also an important mechanism for oxoglaucine-mediated TGFβ signaling suppression, as well as anti-fibrogenic effects.

## 3. Discussion

In the present study, we demonstrated that oxoglaucine, which is known to exert anticancer and anti-inflammatory activities, also inhibits TGFβ-induced fibrosis by affecting the TGF receptor-mediated signaling pathway and ROS generation in hepatocytes (Figure 6E). Importantly, the oxoglaucine concentrations used in this study had anti-fibrogenic activity, without exerting a discernible effect on cell viability. Thus, this study clearly suggests a novel function of oxoglaucine through its effect on Smad2 phosphorylation in Hepa1c1c7 cells and primary hepatocytes.

Our results suggest that oxoglaucine increases TGFβ-induced Smad7 transcript levels. As a negative regulator, Smad7 can decrease cell surface type 1 receptor levels or interfere with complex formation between regulatory Smads and DNA elements [18]. In addition, Smad7 interacts with receptor-regulated Smad (R-Smad) proteins, such as Smad2 and Smad3, upon TGF-β signaling, and it directly inhibits R-Smad activity [43]. Previous studies have shown that TGFβ also activates the AKT pathway in smooth muscle and cancer cells through the TGF receptor [44,45]. However, we found that TGFβ receptor transcript levels were not largely attenuated by oxoglaucine, and the AKT phosphorylation induced by TGFβ was also not significantly affected (Figure 2A). Thus, rather than regulating receptor expression, another mechanism for the anti-fibrogenic effect of oxoglaucine exists. Meanwhile, oxoglaucine largely suppressed AKT phosphorylation in primary hepatocytes, in contrast to Hepa1c1c7 cells (Figure 2B). When we consider activation of the AKT signaling pathway has been found to increase the expression of collagen and other extracellular matrix proteins, oxoglaucine-mediated AKT inhibition, especially in primary hepatocytes, could contribute to the reduction of fibrosis. To prove this possibility, chromatin binding of Smad2 to DNA elements or co-IP experiments between Smad7 and Smad2 after oxoglaucine treatment under constitutively active AKT expression should be performed in both Hepa1c1c7 and primary hepatocytes. Additionally, it is not clear how oxoglaucine suppressed ROS levels in hepatocytes. Because mRNA levels of ROS production enzymes were not changed (Figure 5C), perhaps anti-oxidant components are affected by oxoglaucine to reduce TGFβ-induced ROS levels. Consistent with this hypothesis, isoquinoline alkaloids have been reported to have ROS scavenging properties [46].

Autophagy is an evolutionarily conserved pathway that targets long-lived or defective organelles during degradation. Under nutrient deprivation, autophagy supplies energy for the degradation of cellular components. Autophagy is also known to be involved in various diseases, such as cancer, neurodegenerative diseases, and fibrosis [47,48]. Interestingly, a recent study by Zhong et al. demonstrated that oxoglaucine relieves osteoarthritis by activating autophagy, and inhibition of autophagy reverses the inflammation-alleviating effect of oxoglaucine [27]. Inflammation plays a key role in the development and progression of liver fibrosis. Chronic inflammation causes the liver to produce excess collagen, which can impair liver function and ultimately lead to liver failure. Autophagy, on the other hand, is a process by which cells remove damaged parts and reduce inflammation, thereby preventing the development of chronic diseases associated with inflammation, such as liver fibrosis. Several studies have shown that enhancing autophagy can have an anti-inflammatory effect that may a protective effect against liver fibrosis, by promoting the removal of damaged cellular components. Although we also confirmed that oxoglaucine largely inhibited the mRNA levels of inflammatory cytokines (Figure 4A), the potential contribution of autophagy to this effect remains unclear. Given that autophagy-deficient mice (*Atg5^−/−^*) have been reported to exhibit increased hepatic levels of inflammatory cytokines and fibrosis after chronic carbon tetrachloride (CCl_4_) administration [49], it would be interesting to determine whether oxoglaucine regulates inflammation and fibrosis in hepatocytes by affecting autophagy.

As previously mentioned, isoquinoline derivatives and compounds containing isoquinoline motifs have been investigated for their potential effects on liver fibrosis. For example, sanguinarine has demonstrated anti-fibrotic effects in liver fibrosis models [50]. It can suppress the expression of fibrotic markers, such as *α*-SMA and TGFβ1, thereby reducing fibrosis progression. Additionally, tetrahydropalmatine, an isoquinoline alkaloid, has been investigated for its hepatoprotective effects, including its ability to mitigate liver fibrosis. Tetrahydropalmatine inhibits activation of hepatic stellate cells, reduces collagen deposition, and attenuates inflammation in experimental models of liver fibrosis [51]. Based on database analysis, chemical structures between tetrahydropalmatine (THP) and glaucine are relatively similar, differing only by the presence of an extra double bond in glaucine (ChEBI Search, Tanimoto Score: 0.82). Interestingly, L-THP has been shown to exert anti-fibrotic effects by inhibiting autophagy and the TGFβ1/Smad pathway in hepatic fibrosis in mice and cell models [51]. While these findings are promising, it is important to note that research on isoquinoline compounds for liver fibrosis is still in its early stages, and further studies are needed to validate their effectiveness and mechanism of action.

In summary, we demonstrated that oxoglaucine significantly suppresses TGFβ-induced hepatic fibrosis, and that regulation of Smad7 levels is an important factor in the inhibition of Smad2 phosphorylation. Furthermore, inhibition of ROS generation by oxoglaucine contributes to the anti-fibrogenic activity. Since TGFβ pathway-targeting reagents have been shown to have therapeutic effects on liver fibrosis, oxoglaucine appears to be a potential therapeutic drug for alleviating fibrosis. Nevertheless, further in vivo investigations are warranted to provide further support for our results.

## 4. Materials and Methods

### 4.1. Cell Culture and Material Treatment

Murine hepatoma (Hepa1c1c7) cells were purchased from the American Type Culture Collection (ATCC). The Hepa1c1c7 cells were maintained in Dulbecco modified Eagle’s medium (DMEM) supplemented with 10% fetal bovine serum (FBS) and 1% penicillin/streptomycin. Human hepatic stellate cells (LX-2) were a kind gift of Prof. Seonghwan Hwang (College of Pharmacy, Pusan National University), and cultured in high-glucose Dulbecco modified Eagle’s medium (DMEM) supplemented with 2 mM L-Glutamine and 10 % FBS (Gibco). The cells were maintained at 37 °C with 5% CO_2_ in a humidified chamber. For dose-dependent treatments, oxoglaucine was pretreated at specific doses for 24 h. Recombinant TGFβ was purchased from Abcam (Cambridge, MA, cat. no. ab5003). Oxoglaucine was purchased from ChemFaces (cat. no. CFN90508).

### 4.2. Isolation of Primary Hepatocyte

Hepatocytes were isolated from adult male C57BL/6 mice using a two-step collagenase perfusion method, as previously described [52]. Briefly, under anesthesia, the peritoneal cavity was opened, and the liver was perfused in situ via the portal vein for 4 min at 37°C with calcium–magnesium (CM)-free Hank’s Balanced Salt Solution (HBSS) buffer for 7 min followed by CM-free Hank’s balanced salt solution (HBSS) buffer containing type IV collagenase. The liver was then removed and gently minced, and the released cells were dispersed in DMEM (low glucose) containing 5% fetal bovine serum (FBS) and 1% penicillin/streptomycin. The solution containing the mixed cells and debris was passed through a 100 µm cell strainer. Hepatocytes were inoculated into collagen-coated plates (5 × 10^5^ cells/well in 6-well plates and 1.25 × 10^5^ cells per well in 24-well plates) in Williams’ Medium E with serum free, 1% penicillin/streptomycin, and 0.1 μM dexamethasone. All animal experiments were performed according to the National Institutes of Health Guide for the Care and Use of Laboratory Animals and protocols approved by the Pusan National University-Institutional Animal Care and Use Committee (PNU-IACUC; approval no. PNU-2021-3156).

### 4.3. WST1 Assay

For the WST1 assay, Hepa1c1c7 cells were seeded in 96-well culture plates (2 × 10^4^ cells/well) and cultured for 24 h, after which the cells were exposed to various doses of oxoglaucine. After discarding the incubation medium, the cells were treated with WST1 reagent (Sigma-Aldrich, St. Louis, MO, USA, cat. no. 5015944001) and dissolved in growth medium for 1 h in a CO_2_ incubator at 37 °C. After discarding the solution, 100 μL DMSO was added to each well, and the plate was vortexed for 10 min. The absorbance of the assay solution was measured at a wavelength of 540 nm.

### 4.4. Fluorescence-Activated Cell Sorting (FACS) Assay

To measure apoptosis, Hepa1c1c7 cells were maintained in 6-well culture dishes and incubated with the indicated concentrations of oxoglaucine for 24 h. After trypsinization, the cells were detached and collected by centrifugation (1500 rpm for 5 min). The harvested cells were incubated with FITC-Annexin V (BD Biosciences, San Jose, CA, USA, cat. no. 556419) and 7-ADD (BD Biosciences, San Jose, CA, cat. no. 559925) in the dark for 20 min. The proportions of living and apoptotic cells were determined by flow cytometry (FACS Aria; BD Biosciences, San Jose, CA). 5-Fluorouracil (Sigma-Aldrich, St. Louis, MO, cat. no. F6627-1G) was used as a positive control. For measuring ROS production, the cells were stained with 50 µM 2,7-Dichlorofluoroscin Diacetate (DCFDA, Sigma-Aldrich, St. Louis, MO, cat. no. 287810) solution for 20 min at 37 °C according to the instructions.

### 4.5. RNAi Experiment

Scrambled siRNA and siRNAs against Smad7 (sense: 5′-GAGGCTGTGTTGCTGTGAA-3′, antisense: 5′-TTCACAGCAACACAGCCTC-3′) were purchased from Bioneer Corporation (Daejeon, Republic of Korea). According to the manufacturer’s protocol for siRNA transfection, 12.5 μL of Opti-MEM™ containing 10 μM siRNA was added to 12.5 μL of Opti-MEM™ containing 2 μL of Lipofectamine™ 2000 (Invitrogen, Carlsbad, CA, USA), after incubation for 5 min at room temperature. After gentle mixing, the siRNA-Lipofectamine™ mixture was incubated at room temperature for 15 min, then added to the Hepa1c1c7 cells. The final concentration of the siRNA used in the silencing experiment was 50 nM.

### 4.6. Immunoblotting

The following antibodies were used for immunoblotting: anti-caspase 3 (Cell Signaling #9662S), anti-cleaved caspase 3 (Cell Signaling #9661S), anti-phospho-Smad2 (Ser465/467) (Cell Signaling #3108S), anti-Smad2 (Cell Signaling #5339S), anti-phospho-p44/42 MAPK (Thr202/Tyr204) (Cell Signaling #4370S), anti-phospho-Akt (Ser473) (Cell Signaling #9271S), anti-phospho-AMPK (Thr172) (Cell Signaling #2535S), anti-phospho-acetyl-CoA carboxylase (Ser79) (Cell Signaling #3661S), anti-collagen type I (Invitrogen #PA5-29569), and anti-TGF beta Receptor I (TGFBR1) (Abclonal, #A0708). Immunoblotting was performed by harvesting the treated cells and isolating total proteins. To prepare total cell lysates, the cells were washed with cold PBS and then lysed in cold lysis buffer (40 mM HEPES, pH 7.5, 120 mM NaCl, 1 mM EDTA, 10 mM pyrophosphate, 10 mM glycerophosphate, 50 mM NaF, 1.5 mM Na3VO4, 1 mM PMSF, 5 mM MgCl_2_, 0.5% Triton X-100, and protease inhibitor cocktail). The lysates were sonicated briefly, denatured by heating for 5 min at 95 °C, subjected to SDS-PAGE (9% acrylamide gels), followed by electrophoretic transfer to nitrocellulose membranes. After blocking with 5% non-fat milk in Tris-buffered saline containing 0.05% Tween 20 (TBS-T, pH 7.6), the membranes were incubated overnight at 4 °C with the primary antibodies. After washing the membranes three times with TBS-T, the blots were incubated with HRP-conjugated secondary antibody, washed three times with TBS-T, and protein bands were detected by enhanced chemiluminescence (ECL system; GE Healthcare, Chicago, IL, USA). Densitometric analysis was performed using ImageJ software (ver 1.51).

### 4.7. Quantitative RT-PCR

Total RNA was extracted using TRIzol^®^ reagent (Invitrogen, Carlsbad, CA), and cDNA was reverse-transcribed from 3 μg of total RNA using a High-Capacity cDNA Reverse Transcription Kit (Applied Biosystems, Waltham, MA, cat. no. 4368813). PCR amplification mixtures (20 μL) were prepared by mixing 10 μL of 2× SYBR™ Green PCR Master Mix (Applied Biosystems, Waltham, MA, cat. no. 4364344), 2 μL of primer mix (1 μM forward and 1 μM reverse primers), and 8 μL of diluted cDNA templates. Real-time quantitative PCR was performed on a QuantStudio™ Real-Time PCR system (Applied Biosystems, Waltham, MA) using the following conditions: 95 °C for 1 min, followed by 40 amplification cycles at 95 °C for 15 s, 60 °C for 15 s (annealing), and 72 °C for 30 s. After amplification, a melting curve analysis was performed according to the manufacturer’s instructions. The oligonucleotide sequences for this study were as follows: *α-SMA*, forward 5′- TGCTGACAGAGGCACCACTGAA-3′, reverse 5′- CAGTTGTACGTCCAGAGGCATAG-3′; *Fn1*, forward 5′-CCCTATCTCTGATACCGTTGTCC-3′, reverse 5′- TGCCGCAACTACTGTGATTCGG-3′; *Timp1*, forward 5′- TCTTGGTTCCCTGGCGTACTCT-3′, reverse 5′-GTGAGTGTCACTCTCCAGTTTGC-3′; *Col1a1*, forward 5′-CCTCAGGGTATTGCTGGACAAC-3′, reverse 5′- CAGAAGGACCTTGTTTGCCAGG′; *Smad7*, forward 5′-GTCCAGATGCTGTACCTTCCTC-3′, reverse 5′- GCGAGTCTTCTCCTCCCAGTAT-3′; *Il-6*, forward 5′- TCGTGGAAATGAGAAAAGAGTTG-3′, reverse 5′-AGTGCATCATCGTTGTTCATACA-3′; *Ccl2*, forward 5′-ACAAGAGGATCACCAGCAGC-3′, reverse 5′- GGACCCATTCCTTCTTGGGG-3′; *Tnfα*, forward 5′- CTGAGGTCAATCTGCCCAAGTAC-3′, reverse 5′- CTTCACAGAGCAATGACTCCAAAG-3′; *Nox4*, forward 5′-CGGGATTTGCTACTGCCTCCAT-3′, reverse 5′-GTGACTCCTCAAATGGGCTTCC-3′. *Gapdh*, forward 5′-CAAGGTCATCCATGACAACTTTG-3′, reverse 5′-GGCCATCCACAGTCTTCTGG-3′. Expression levels were measured by ΔΔCT analysis.

### 4.8. Statistical Analysis

All data were evaluated using GraphPad software (Version 8.01, San Diego, CA, USA) and are expressed as means ± the S.E.M. Data for multiple variable comparisons were analyzed by a one-way ANOVA with Tukey post hoc test, and comparisons between two groups were analyzed by unpaired two-tailed Student’s *t*-test, with a *p* < 0.05.

## Data Availability

Data is contained within the article or Supplementary Material.

## Supplementary Materials

The following supporting information can be downloaded at: https://www.mdpi.com/article/10.3390/molecules28134971/s1, Figure S1: Oxoglaucine alone did not affect fibrogenic gene expression or intracellular ROS levels in hepatocytes.
