# Supplementary material for: Oxoglaucine Suppresses Hepatic Fibrosis by Inhibiting TGFβ-Induced Smad2 Phosphorylation and ROS Generation
Source: Molecules. 2023 Jun 24;28(13):4971. doi: 10.3390/molecules28134971 (PMC10343928; doi:10.3390/molecules28134971)
Supplement: Supplementary file 1 [file molecules-28-04971-s001.zip › molecules-2353299-supplementary.pdf]

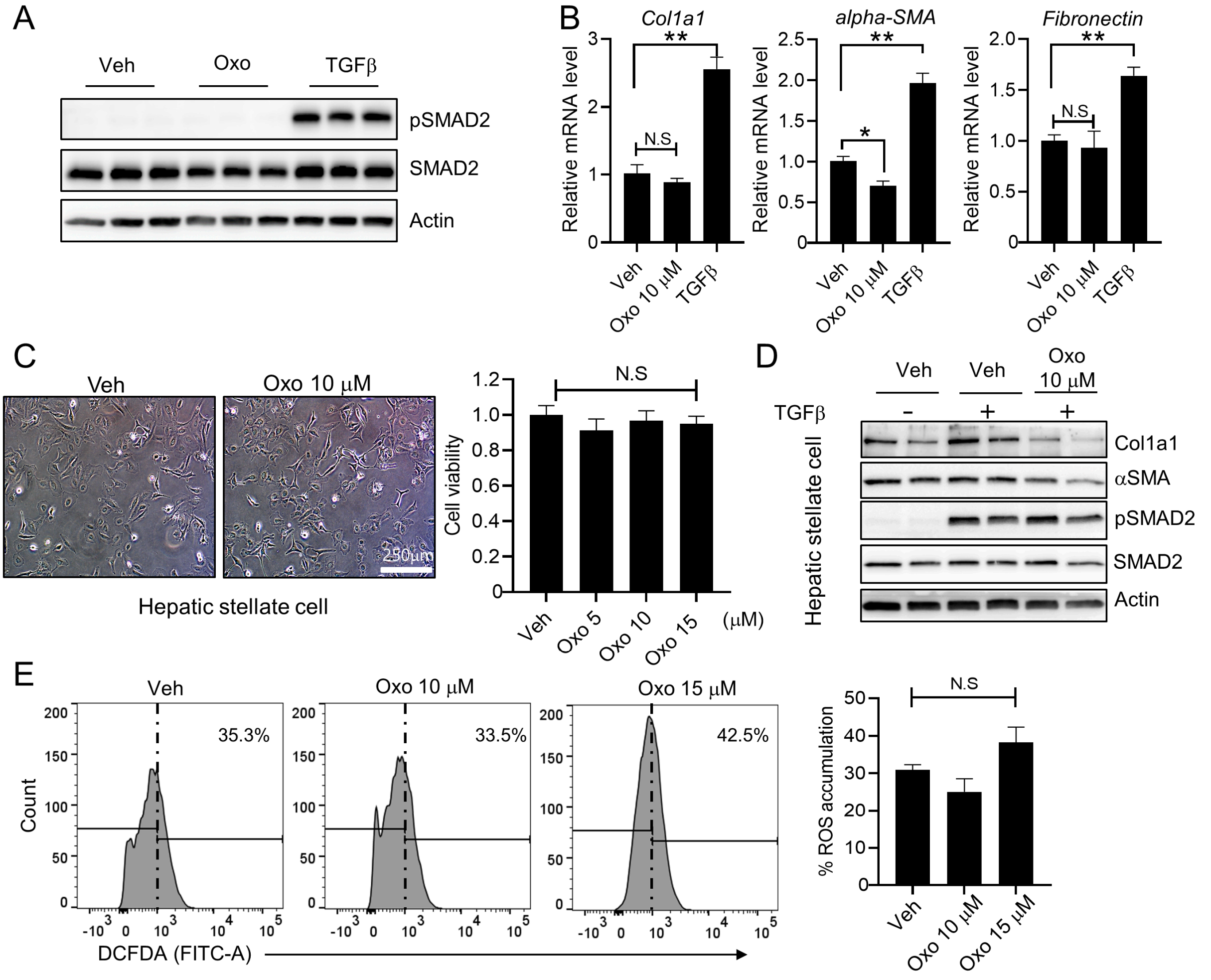

Supplemental Figure S1. Oxoglaucine alone did not affect fibrogenic gene expression or intracellular ROS levels in hepatocytes. Oxoglaucine (10 μM) treatment for 24 h without TGFβ. Smad2 phosphorylation (A) and *Col1a1*, *αSMA*, and *Fn1* transcript levels (B) in Hepa1c1c7 cells. (C) Stellate cells were treated with oxoglaucine for 24 hour. Morphology (left) and viability (right) of LX-2 cells were confirmed. (D) LX-2 cells were treated with the indicated concentrations of oxoglaucine for 24 h followed by TGFβ (5 ng/mL) stimulation for an additional 6 h. The lysates were subjected to immunoblot assay using indicated primary antibodies. (E) Measuring ROS levels using FACS analysis with indicated conditions. Results are represented as mean values from three independent experiments. Values are shown as ± S.E.M; * $p<0.05$, ** $p<0.01$. N.S indicates not significant.
